# Development and validation of a sensitive LC-MS/MS method for determination of intracellular concentration of fluconazole in *Candida albicans*

**DOI:** 10.3389/fmicb.2022.1007576

**Published:** 2022-10-05

**Authors:** Xiaofei Wang, Xiaojuan Wang, Tongkai Cai, Yulin Qin, Ling Li, Yuanying Jiang, Bing Han, Yongbing Cao

**Affiliations:** ^1^Institute of Vascular Disease, Shanghai TCM-Integrated Hospital, Shanghai University of Traditional Chinese Medicine, Shanghai, China; ^2^School of Pharmacy, Naval Medical University, Shanghai, China; ^3^Department of Pharmacy, Mudanjiang First People’s Hospital, Mudanjiang, China; ^4^Department of Pharmacy, Minhang Hospital, Fudan University, Shanghai, China

**Keywords:** fluconazole, *Candida albicans*, liquid-liquid extraction, LC-MS/MS, intracellular concentration

## Abstract

Systemic candidiasis is the fourth leading cause of healthcare-associated infections worldwide. The combination therapy based on existing antifungal agents is well-established to overcome drug resistance and restore antifungal efficacy against drug-resistant strains. In this study, a simple and sensitive liquid chromatography with tandem mass spectrometry (LC-MS/MS) method was developed to quantify the intracellular fluconazole (FLC) content in the opportunistic human fungal pathogen *Candida albicans*. The cell lysates were prepared by lysing *C. albicans* cells with Precellys homogenizers and FLC was extracted with methylene chloride. The entire extraction approach was simple, precise and reliable. The extracts were separated on a Zorbax SB-C18 column using a mobile phase of acetonitrile (solvent A) and deionized water plus 0.1% formic acid. FLC and ketoconazole (KCZ, internal standard) were monitored in positive mode using electrospray ionization source. The multiple reaction monitoring transitions (precursor to product) were monitored for FLC m/z 307.1 → 238.2 and for the internal standard KCZ m/z 531.2 → 489.1. The linear for this method were in the range from 5.0 to 1000.0 ng/mL. The precision and accuracy of the samples were relative standard deviations (RSD) < 1.0% for intra-day and RSD < 0.51% for inter-day. The overall recovery of FLC from samples was higher than 77.61%. Furthermore, this method was successfully applied and validated in 36 clinical isolated strains. Taken together, we established a highly accurate, efficient, and reproducible method for quantifying the intracellular content of FLC in *C. albicans*.

## Introduction

*Candida albicans* (*C. albicans*) is one of the most common commensal fungal species located in the gastrointestinal and reproductive tracts of healthy individuals, causing both mucosal and systemic infections in immunocompromised individuals (Brown et al., 2012; de Oliveira Santos et al., 2018; Quindós et al., 2018). Systemic candidiasis is a serious healthcare-associated infection in Europe and US, and associated with high mortality rates (40%) among hospitalized patients, particularly in individuals with hematological malignancies, undergoing major surgery, cytotoxic chemotherapy, and organ transplantation (Magill et al., 2014; Bongomin et al., 2017; Pappas et al., 2018; Hou et al., 2022). Currently, fluconazole (FLC), a highly selective inhibitor of fungal cytochrome P-450 sterol C-14 alpha-demethylation, is the most widely administered antifungal for treating invasive, life-threatening fungal infections (Robbins et al., 2017; Revie et al., 2018). However, high administration frequency and long duration treatment of FLC contribute to the rising number of drug resistant *C. albicans* worldwide (Berkow and Lockhart, 2017; Campitelli et al., 2017; Pristov and Ghannoum, 2019). The primary mechanism of drug resistance is the reduction of intracellular accumulation of azole in *C. albicans*, due to reduced drug uptake or increased drug efflux (Arendrup and Patterson, 2017; Wiederhold, 2017). Therefore, the development of new therapeutic agents to restore *C. albicans* susceptibility to FLC is an effective strategy for the treatment of fungal infections.

The use of drug combination therapy has been successfully implemented for difficult-to-treat infections, such as malaria, tuberculosis, and AIDS (Robbins et al., 2017). Indeed, combination therapy represents an effective method to overcome the emergence of drug-resistant fungi and decrease toxicity (Zacchino et al., 2017; Ribeiro de Carvalho et al., 2018). However, many studies have shown results that range from antagonism to synergy effects due to the different concentrations of each drug combination (Johnson et al., 2004; Campitelli et al., 2017; Tome et al., 2018). The discrepancy may be caused by different measurements of intracellular drug content. In order to accurately detect the intracellular concentration, high performance liquid chromatography with tandem mass spectrometry (HPLC–MS/MS) was used to measure the intracellular FLC levels in *C. albicans*. At present, several fast HPLC–MS/MS methods have been validated and reported for monitoring the antifungal drug concentration in plasma or other body liquid, including FLC, itraconazole, and other antifungal agents (Van De Steene and Lambert, 2008; Tang et al., 2010; Zhang et al., 2011; Alebic-Kolbah and Modesitt, 2012; Beste et al., 2012; Zgoła-Grześkowiak and Grześkowiak, 2013; de Moraes et al., 2014; Wadsworth et al., 2017; Różalska et al., 2018; Xiang et al., 2018). Actually, quantitative analysis of FLC in *C. albicans via* HPLC-MS/MS has not been reported. In this study, we developed a specific, reliable and sensitive liquid chromatography with tandem mass spectrometry (LC-MS/MS) method for determining the intracellular levels of FLC in *C. albicans*.

## Materials and methods

### Strains and growth conditions

The FLC-resistant *C. albicans* strains NOs. 100 and 103 were obtained from Changhai hospital (MIC_80_ > 1,024 μg/mL). In addition, 36 clinical isolated strains of FLC-resistant or FLC-sensitive *C. albicans* were obtained from Tianjin University. All strains were stored with 15% glycerol at −80^°^C and subcultured on sabouraud dextrose agar (SDA) plates (4% dextrose, 1.8% agar, and 1% peptone) at 30°C. Exponentially growing *C. albicans* cells were routinely grown in yeast-peptone-dextrose (YPD) liquid medium (2% peptone, 2% dextrose, and 1% yeast extract) at 30°C in a shaking incubator overnight for the following experiments.

### Chemicals and reagents

FLC and ketoconazole (KCZ) (> 99.0%) were purchased from Sigma-Aldrich (St Louis, MO, USA). Acetonitrile was liquid chromatography (LC) grade and purchased from Merck (Darmstadt, Germany). HPLC-grade formic acid was purchased from Tedia Company (Fairfield, OH, USA). Dichloromethane, sodium hydroxide and dimethyl sulfoxide were purchased from Shanghai Chemical Reagent Company (Shanghai, China). Deionized water was prepared from Milli-Q water purifying system (Millipore Corporation, Bedford, MA, USA). Methanol was purchased from Merck (Darmstadt, Germany).

### Internal standards and calibration standards

FLC and KCZ were weighed and solved in methanol at a concentration of 1.00 mg/mL, respectively. Working solution of FLC (100, 10, and 1.0 μg/mL) was prepared by the dilution of the stock solution. The stock solution of KCZ and H_2_O were mixed to obtain working solution at a concentration of 100 μg/mL. Stock solutions were stored at −70°C and the standard solutions were prepared immediately before use.

### Liquid chromatography with tandem mass spectrometry conditions

The LC-MS/MS analysis was performed using the triple quadrupole mass spectrometer (Aglient 6410A, Santa Clara, USA) in the selected reaction monitoring (SRM) mode. The columns were chromatographic column Zorbax SB-C18 column (3.5 μm, 100 mm × 2.1 mm i.d., Agilent, Palo Alto, CA). The mobile phase was composed of acetonitrile (solvent A) and 0.1% formic acid in distilled deionized water (solvent B), a 40:60 (v/v) mixture of solvent A and B. Flow rate was 0.3 mL/min; run time was 2.3 min. The column temperature was maintained at 35°C and the injection volume was 10 μL.

The LC-MS/MS conditions were as follows: electrospray ionization (ESI) in positive mode; capillary voltage, 4,000 V; vaporizer temperature, 40°C; atomization gas (nitrogen) pressure, 0.276 MPa; desolution gas (nitrogen) temperature, 350°C, flow rate, 10.0 L/min. The collision gas (high purity nitrogen) pressure was 0.1 MPa. Half width of the mass spectrum was 0.7 amu. The mass spectrometer was operated under multiple reaction monitoring (MRM) modes with collision energy of 18 eV for FLC and 40 eV for KCZ. The following MRM transitions (precursor to product) were monitored for FLC m/z 307.1 → 238.2 and for the internal standard (IS) KCZ m/z 531.2 → 489.1 (Table 1).

**TABLE 1:** Optimized MRM (multiple reaction monitoring) parameters for FLC and KCZ.

	Precursor ion (m/z)	Fragmentor energy (V)	Collision energy (eV)	Product ion (m/z)
FLC	307.1	80.0	18.0	238.2
KCZ (IS)	531.2	100.0	40.0	489.1

### Sample preparation

#### *Candida albicans* lysates preparation

The logarithmic growth *C. albicans* was harvested and re-suspended to 5 × 10^9^ CFU/mL with YPD liquid medium. FLC stock solution (1 mg/mL) were added to the suspension. The final concentration of *C. albicans* was adjusted to 5 × 10^7^ CFU/mL and FLC concentration was diluted to 16 μg/mL. The mixture was incubated at 30°C with agitation at 200 rpm for 16 h. Subsequently, *C. albicans* cells were collected by centrifuging the suspension for 30 s at 5,000 × *g*. Samples were washed for four times with equivalent volume of the original culture medium and centrifuged to remove residual medium and FLC. After that, the precipitation was resuspended and centrifuged four times at 16,200 × *g* to remove the liquid. 500.0 mg of fungal cells were added to the Eppendorf tube together with a volume of 1.5 mL deionized water and 180.0 μL 0.5 mm glass beads, 180.0 μL 0.1 mm glass beads, 180.0 μL 1 mm ceramic bead and two 3 mm ceramic beads. All samples were crushed in a Precellys 24 biological sample homogenizer (Bertin Technologies, Montignyle-Bretonneux, France) with the following protocol: 6,500 rpm/min, 30 s, 3 times, interval of 30 s; 3 cycles, interval of 5 min. All samples were kept on ice during the circulation interval. *C. albicans* lysates solution was harvested after centrifugation.

#### *Candida albicans* lysates extraction

The *C. albicans* lysates solution (100 μL) was added into a centrifuge tube containing internal standard solution, 20.0 μL KCZ (1.0 μg/mL) and 10.0 μL NaOH (20.0 μg/mL). After vortexing for 30 s, 3.0 mL of dichloromethane (CH_2_Cl_2_) was finally added and mixed thoroughly. The liquid system was divided into two layers after 10 min centrifugation at 9,982 × *g.* Next, 2.4 mL of liquid was removed from the lower layer and the CH_2_Cl_2_ phase was transferred into a clean centrifuge tube and evaporated to dryness in the centrifugal thickener (35°C heat, heat time: 50 min, run time: 200 min). After that, 80 μL of mobile phase [acetonitrile: 0.1% formic acid = 40:60 (v/v)] was added to the evaporated sample tubes and vortex-mixed for 1 min. The liquid was transferred to a new 1.5 mL centrifuge tube and centrifuged at 21,000 × *g* for 10 min. Following, the supernatant was then transferred to the vial (containing the inner tube) for LC-MS/MS analysis.

### Validation of the liquid chromatography with tandem mass spectrometry method

The validation including selectivity, matrix effect, linearity, precision, and accuracy, the limits of detection (LOD) and quantification (LOQ), extraction recovery and stability were conducted in accordance with the regulatory guidelines on bioanalytical method validation.

#### Selectivity

The product ions of m/z 307.1→238.2 (FLC) and m/z 531.2→489.1 (KCZ) were analyzed by full scanning, and the fragment ions were used as product ions monitored during the quantitative analysis.

#### Linearity

The linearity was investigated by analyzing a seven-point (5.0, 10.0, 50.0, 100.0, 200.0, 500.0, and 1,000.0 ng/mL) calibration curve of FLC in *C. albicans* lysate in triplicate. Calibration curve were constructed by plotting the peak area ratios of FLC/internal standard vs. the concentrations of FLC in *C. albicans* lysate, using weighted (1/*c*^2^) least squares linear regression. Slope, intercept, and correlation coefficient were calculated as regression parameters by using a 1/x weighed linear regression.

#### Precision and accuracy

Precision and accuracy were assessed in within-run (repeatability and accuracy in 1 day) and between-run conditions (intermediate precision and intermediate accuracy). Precision was calculated as relative standard deviations (RSD) in percentage, whereas accuracy was calculated as relative error (RE) in percentage, between a nominal concentration value in the calibration sample and a concentration obtained from the calibration curve. Low, medium and high concentrations of FLC (10.0, 100.0, and 500.0 ng/mL, respectively) were used to analyze intra-day precision and accuracy. Moreover, five replicates of each sample at low, medium and high concentration levels were analyzed on the same day. The assay was performed in three consecutive days to evaluate inter-day precision and accuracy.

#### Limit of detection and limit of quantification

LOD and LOQ were determined by spiking a decreasing concentration of the mixed stock solution into blank *C. albicans* lysate. The LOD was defined as the lowest concentration point at which the instrument exhibits a signal-to-noise (S/N) ratio equal to 3. The LOQ was defined as the lowest concentration reliably quantified and fulfilled the criteria of not exceeding ± 20% mean relative error (MRE) and < 20% RSD.

#### Extraction recovery and matrix effect

The samples were spiked with blank *C. albicans* lysate and prepared with FLC final concentrations of 10.0, 100.0, and 500.0 ng/mL. The extractions of the samples containing different concentrations of FLC were prepared as described in section “*Candida albicans* lysates extraction.” Then, the samples extractions and different concentrations of FLC standard solution were detected by LC-MS/MS. The extraction recovery rates of samples containing different concentrations of FLC were obtained by comparing the chromatographic peak areas of the same concentration of extraction sample and the FLC standard solution.

In order to develop a reliable and reproducible method, the matrix effect was also investigated. The matrix effect was evaluated by the following experiment. Triplicates of QC samples at three levels of FLC and IS were added into 100 μL *C. albicans* lysates and water separately, and then the spiked samples were pretreated with exactly the same procedure as described in *Candida albicans* lysates preparation section. Then, the samples extractions were detected by LC-MS/MS. Comparison of the chromatograms of the blank and the spiked *C. albicans* lysates was used to assay the selectivity of the method. The matrix effect was determined by observing the signal of the chromatogram.

#### Stability

Stability of FLC in extracted samples was evaluated at three concentrations (high, medium, low) in triplicate under different conditions, including three freeze (−80°C)/thaw (25°C) cycles, 1-month storage in −20^°^C or 6 h storage at room temperature. The post-preparative stability was also evaluated by keeping samples in mobile phase at room temperature for 24 h.

### Statistical analysis

GraphPad Prism 9 was applied to analyze the statistical significance of data. At least three independent replicates were conducted for all experiments unless otherwise stated and *P* < 0.05 was considered statistically significant. For multiple comparisons, *P*-values were calculated by using one-way analysis of variance (ANOVA). For single comparison, *P*-values were calculated by using two-tailed Student’s *t*-test.

## Results

### Liquid chromatography with tandem mass spectrometry optimization

**FIGURE 1:**
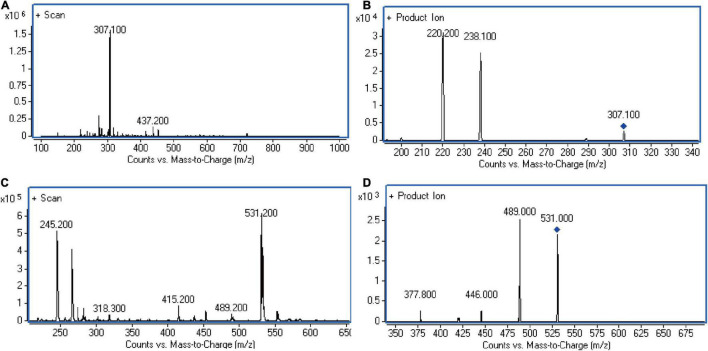
LC-MS/MS chromatograms. **(A)** Full-scan of FLC in standard solution. **(B)** Product ion of FLC in standard solution. **(C)** Full-scan of KCZ in internal standard solution. **(D)** Product ion of KCZ in internal standard solution. The representative results of three independent experiments are shown.

The suitable internal standard was selected to correct the errors that might occur in each process of sample pretreatment, and it is especially important to correct errors caused by instrument instability when mass spectrometry was used as a detector. Internal standards are usually required to have same or similar structural and physical and chemical properties as the analyte. Therefore, KCZ was chosen as the internal standard of this experiment. The structure of KCZ has a certain similarity compared with FLC. In the positive ESI mode, the analyte and IS formed predominately protonated molecular ions [M + H]^+^ in full scan mass spectra. Figure 1 displayed product ion spectra of [M + H]^+^ ions from two compounds. Two fragment ions were observed in the product ion spectra. The major fragment ions at m/z 307.1→238.2 and m/z 531.2→489.1 were chosen in the MRM acquisition for FLC and IS, respectively. Moreover, KCZ was not detected in *C. albicans* lysate. Hence, KCZ met the conditions as an internal standard.

### Sample preparation

In order to make the intracellular FLC fully release from *C. albicans*, efficient and flexible Precellys 24 biological sample homogenizer offered by Bertin technologies was used for grinding samples prior to analysis. The method could make the cell wall broken completely and conducive to the extraction and separation of FLC from the *C. albicans* cells. Moreover, the method was simple and efficient. At the beginning of the study, liquid-liquid extraction solvents such as methyl tertiary butyl ether, ethyl acetate or dichloromethane were investigated to process biological samples. After dissolved with the mobile phase [A phase is acetonitrile, B phase is water (containing 0.1% formic acid), A:B = 40:60 (v/v)], the liquid samples were detected by LC-MS/MS. Our results showed that the extraction recovery rate significantly increased to about 81%, and more importantly, samples obtained were clean with less impurities when dichloromethane was used with a small amount of sodium hydroxide (Table 2). Indeed, when samples were re-dissolved and liquid samples were injected after the mobile phase, ideal peak shapes with highest extraction recovery rate (∼ 81%) were observed. Therefore, liquid-liquid extraction to treat *C. albicans* lysate samples was used with dichloromethane and a small amount of sodium hydroxide.

**TABLE 2:** Extract recovery of FLC (*n* = 3).

Theoretical concentration (%)	100.00
Methyl tert-butyl-ether (%)	30.51 ± 4.45
Ethyl acetate (4 μL of 5 mol sodium hydroxide, %)	67.28 ± 7.76
Ethyl acetate (40 μL of 2 mol ammonia water, %)	44.19 ± 6.51
Dichloromethane (40 μL of 2 mol ammonia water, %)	65.28 ± 4.13
Dichloromethane (4 μL of 5 mol sodium hydroxide, %)	81.30 ± 8.96

### Validation

#### Selectivity

**FIGURE 2:**
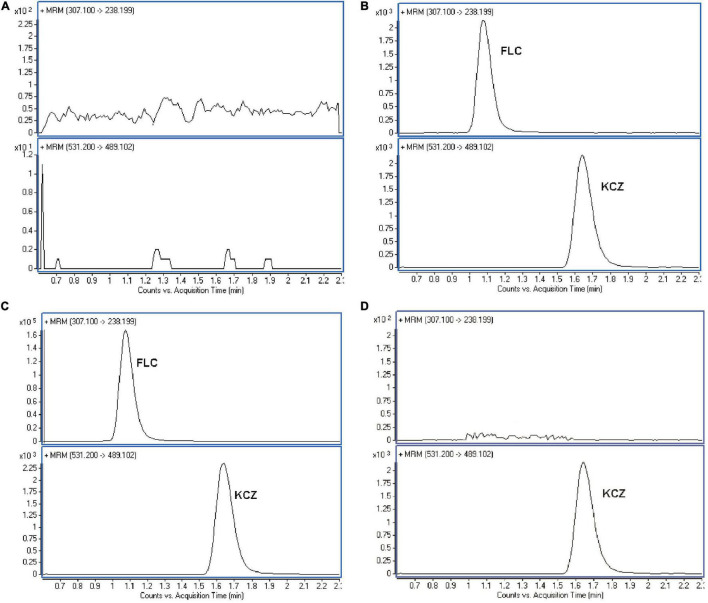
Representative MRM chromatograms of FLC in *C. albicans* lysate. **(A)** Blank sample. **(B)** Blank sample with FLC and KCZ (internal standard). **(C)** The testing *C. albicans* lysate. **(D)** Blank sample with KCZ (internal standard). The representative results of three independent experiments are shown.

The LC–MS/MS detection has high selectivity that only ions generated from the selected precursor ions can be monitored. Comparison the chromatograms of the blank and the spiked *C. albicans* lysate, the retention times of the analytes and the IS has no significant interference (Figure 2). The retention time of FLC and KCZ were 1.06 and 1.49 min, respectively. The endogenous impurities in *C. albicans* lysates did not interfere with determination of FLC and KCZ, indicating that the method was specific for FLC analysis in *C. albicans.*

#### Linearity

**FIGURE 3:**
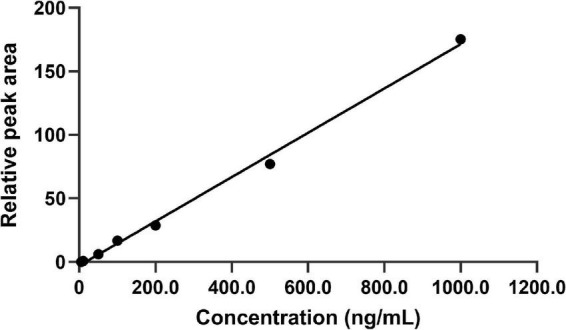
Plot of back-calculated concentrations of calibration standards of FLC. The standard curve equation was Y = 0.1742C–2.8763 (*n* = 5). The limits of detection for this method were in the range from 5.0 to 1000.0 ng/mL. The representative results of three independent experiments are shown.

The calibration curves were linear ranging from 5.0 to 1000.0 ng/mL with the correlation coefficient was 0.9963. The results showed that the standard curve equation of FLC in *C. albicans* lysates solution was Y = 0.1742C–2.8763 (*n* = 5). Moreover, the LOQ was 5.0 ng/mL. The standard curve of FLC in *C. albicans* lysate is shown in Figure 3.

#### Precision and accuracy

Precision and accuracy were determined by replicating the analyses of three known concentrations over the calibration curve on 3 different days. The intra-day accuracy ranged from −12.9 to 10.8%, and the precision ranged from 1.00 to 1.54% (Table 3). The inter-day accuracy and precision were −12.6 ∼11.6% and 0.51 ∼ 0.85%, respectively. The deviation of the measured concentrations from the true value was reached ± 15% of nominal (theoretical) concentrations. These results demonstrated that the method was reproducible and accurate.

**TABLE 3:** Intra-day and inter-day assay precision and accuracy of FLC in *C. albicans* lysate samples (*n* = 5).

Conditions	Concentration (ng/mL)	Accuracy (%)	Precision (%)
Intra-day	10.0	−12.9	1.00
	100.0	10.8	1.46
	500.0	−11.1	1.54
Inter−day	10.0	−12.6	0.51
	100.0	11.6	0.68
	500.0	−10.9	0.85

#### Extraction recovery and matrix effect

We evaluated the extraction recoveries of FLC at three different concentrations (10.0, 100.0, and 500.0 ng/mL). As shown in Table 4, the extraction relative recoveries of low, medium, and high concentrations were 87.10 ± 0.09%, 110.82 ± 1.62%, and 88.87 ± 13.64%, respectively. High extraction recoveries were observed in *C. albicans* lysate samples, suggesting that extraction efficiency ensured FLC stability. The results of matrix effect experiments showed that there was no significant difference between the peak areas of samples prepared from *C. albicans* lysate and water, indicating that no co-eluting unknown compounds had apparent effect on the ionization of analytes and IS (Figure 2).

**TABLE 4:** Recovery of FLC in *C. albicans* lysate samples (*n* = 5).

Drug	Nominal concentration (ng/mL)	Measured concentration (ng/mL)	Relative recovery (%)	RSD (%)
FLC	10.0	8.71 ± 0.09	87.10 ± 0.09	1.00
	100.0	110.82 ± 1.62	110.82 ± 1.62	1.46
	500.0	444.36 ± 6.84	88.87 ± 13.64	1.54

#### Limit of detection and limit of quantification

The LOD of FLC was 0.5 ng/mL with an RSD of 2.38%. The present LC–MS/MS method offered an LOQ 5.0 ng/mL with an accuracy of −7.6% in terms of RE and a precision of 5.42% in terms of RSD (*n* = 5). This indicated a highly sensitive method was established.

#### Stability

FLC remained stable during sample preparation and storage. The stability of FLC was evaluated under various conditions and summarized in Table 5. The relative deviation of samples undergoing the three freeze (−80^°^C)-thawed (25^°^C) cycle was RSD < 2.5%, RSD < 2.1%, and RSD < 3.4% for samples in low, medium and high-quality control samples, respectively. In addition, RSD < 3.8%, RSD < 9.5%, and RSD < 6.1% was observed for samples treated in the mobile phase at room temperature for 24 h in low, medium, and high quality control samples. Moreover, the content of FLC was no significant decreased while *C. albicans* lysate samples has been stored at −20^°^C for 30 days. Relative recovery was more than 94.69% in all the quality control samples, indicating that FLC was stable in *C. albicans* lysate during the whole analytical process.

**TABLE 5:** Freeze thawing of FLC in *C. albicans* lysate samples (*n* = 3).

Storage conditions (*n* = 3)	Nominal concentration FLC (ng/mL)	Calculation concentration FLC (ng/mL)		
		Mean	Relative recovery (%)	RSD (%)
Pre-preparative stability (25^°^C, 6 h)	10.00	9.84	98.40	3.88
	100.00	103.47	103.47	6.05
	500.00	512.44	102.49	9.59
Pre-preparative stability (25^°^C, 24 h)	10.00	9.88	98.87	3.77
	100.00	94.69	94.69	9.42
	500.00	531.06	106.21	6.05
Long-term storage stability (−20^°^C, 30 days)	10.00	9.97	99.73	3.21
	100.00	101.26	101.26	7.59
	500.00	505.99	101.20	6.96
Three freeze (−80^°^C) and thaw (25^°^C) cycles	10.00	9.60	96.01	2.45
	100.00	110.99	110.99	2.00
	500.00	552.88	110.58	3.36

### Application to clinical *Candida albicans* strains

**FIGURE 4:**
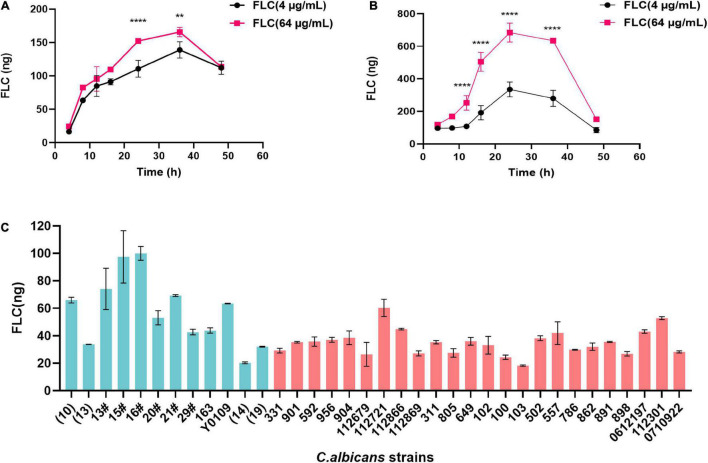
Intracellular levels of FLC in 500.0 mg wet *C. albicans* samples. **(A)** Levels of FLC in samples derived from NO. 103 FLC-resistant *C. albicans* strain at different time points. **(B)** Levels of FLC in samples derived from NO. 100 FLC-resistant *C. albicans* strain at different time points. **(C)** Levels of FLC in *C. albicans* samples after 1-h incubation with 1 μg/mL FLC. The green columns represent FLC-sensitive *C. albicans* strains, and the red columns represent FLC-resistant *C. albicans* strains. Data are mean ± SD from triplicates of one representative experiment of three. **P < 0.01, ****P < 0.0001 [**(A,B)**, one-way analysis of variance (ANOVA)].

The method validated in this study was applied to clinical isolated *C. albicans* strains, including FLC-sensitive and FLC-resistant strains. Figures 4A,B showed the concentrations changes in FLC-resistant strains NO. 100 and NO. 103 treated with 4 and 64 μg/mL FLC, respectively. The concentration of intracellular FLC in *C. albicans* were gradually increased and then decreased during the 48 h detect time, with a maximum level at 24∼36 h. Furthermore, the concentration of FLC was also measured in 36 clinical isolated *C. albicans* that was incubation with FLC at a concentration of 1.0 μg/mL. As shown in Figure 4C, the mean intracellular concentration of fluconazole in FLC-sensitive *C. albicans* strains (green columns) was significantly higher than FLC-resistant *C. albicans* (red columns). However, no major differences in the intracellular FLC concentration were observed between several sensitive strains (strain13, 14, and 19) and the majority of FLC-resistant strains. Although the reason for this discrepancy is unclear, it might result from the different expression of drug efflux genes in *C. albicans* cell wall, including *CDR1*, *CDR2*, and *MDR1* (Kofla et al., 2011; Rocha et al., 2017; Dhasarathan et al., 2021; Xu et al., 2021).

## Discussion

The effective combination-based therapy is a feasible regimen for the majority of refractory infections disease. FLC represents one of the most commonly available antifungal drugs in clinical practice (Lu et al., 2021). Our previous research has shown that the combination of FLC and berberine (BBR) has a significant synergistic against FLC-resistant *C. albicans*, but the synergistic effect was not observed in FLC-sensitive *C. albicans* (Quan et al., 2006). To elucidate whether FLC exerts discrepant effects against *C. albicans* due to intracellular FLC, we performed LC-MS/MS approach to quantify the concentration of FLC in *C. albicans* strains. LC-MS/MS is frequently used as a detector to monitor selected ions and specific fragment ions generated by the ions. Currently, available assays for detecting intracellular FLC include bioassays, gas chromatography assays, and high-performance liquid chromatographic methods. In this study, a sensitive and convenient LC-MS/MS method for the determination of intracellular levels of FLC in *C. albicans* was developed and validated.

Indeed, it is well known that sample extraction plays a particular role in LC analysis, especially for small volume samples requiring purification. The conventional sample preparation approaches of *C. albicans* lysates for LC analysis include protein precipitation, liquid-liquid extraction or solid phase extraction (Diez et al., 2005; Beste et al., 2012). Although drug extraction rate prepared by solid phase extraction is higher and the sample is clean and automated solid phase extraction instrument can achieve rapid pretreatment of large samples, this method is rarely used because of the expensive columns. Compared to protein precipitation and solid phase extraction, liquid-liquid extraction is one of the most commonly used methods for sample pretreatment (Kim et al., 2018). Relatively pure samples can be obtained by using liquid-liquid extraction with low cost, but this method is not suitable to the low extraction rate or instability drugs.

In the present study, a simple sample preparation and extraction protocol including the use of Precellys homogenizers and methylene chloride for FLC extraction were optimized to provide adequate sensitivity, appropriate samples cleanliness, excellent recovery rate. The method was fully validated based on international guidelines and all evaluated parameters met the pre-established criteria (Zimmer, 2014). Moreover, the suitability of the method was applied to 36 clinical isolated *C. albicans* strains.

In order to release the intracellular FLC of *C. albicans*, efficient and flexible Precellys 24 biological sample homogenizer offered by Bertin technologies was applied for grinding samples prior to analysis. Moreover, the sample preparation method was further optimized by using glass beads to homogenize *Candida* cells. The glass beads allowed the cell wall broken completely, facilitating the extraction and isolation of FLC from *C. Candida* cells. The integrity of the *C. albicans* cell was observed under the microscope and the protein content was determined after extraction. Importantly, this extraction method based on the release of FLC from cell breakage was simple and not laborious. It can be used in routine microbiology laboratories to quantify FLC in fungi and improve experimental operation to increase the reproducibility and accuracy.

Internal standard with similar structural and physicochemical properties provides multiple advantages in HPLC-MS/MS bioanalytical process, including reduction of analysis run time, improvement of the intra-injection reproducibility, reduction of matrix and ionization effects (Bergeron et al., 2009). KCZ and FLC have the similar structural and physicochemical properties and belong to the same antifungal drug class. Our results demonstrated that using KCZ as the internal standard was feasible (Table 1). Subsequently, the characteristics of LC-MS/MS approach, including selectivity, linearity, LOD, LOQ, precision, accuracy, extraction recovery, matrix effect, and stability were validated (Tables 2–5 and Figures 1–3).

Furthermore, the established LC-MS/MS method was validated in 36 clinical isolated *C. albicans* strains. According to Figures 4A,B, the concentration of FLC *C. albicans* cells was low at the early stage therapeutic exposures. Specifically, intracellular drug concentrations were gradually increased, reaching the maximum concentration between 24 and 36 h, and then gradually decreased over time. We speculate that the changes of FLC concentration may be due to the logarithmic reproduction of *C. albicans* after 24 h culture, as the most active division and reproduction of *C. albicans*. During this stage, a large number of substances need to be absorbed from the culture medium for reproduction, and thus FLC was more efficiently untaken by *C. albicans*. With the rapid increase in the number of *C. albicans* and the continuous consumption of culture media, *C. albicans* become tolerant to drugs, and the efflux of intracellular drugs continues to increase, resulting in decreased content of intracellular drug after 36 h.

The intracellular content of FLC is not the same among different *C. albicans* strains in the same co-culture time (Figure 4C). The intracellular FLC content of a majority of sensitive *C. albicans* is significantly higher than that of drug-resistant *C. albicans*, which may be due to membrane permeability or the high expression of *CDR1*, *CDR2*, and *MDR1* in FLC-resistant *C. albicans* strains, and the reduction of intracellular azole drug content caused by drug efflux (Kofla et al., 2011; Rocha et al., 2017; Dhasarathan et al., 2021; Xu et al., 2021). However, the results also showed that intracellular FLC content of FLC-sensitive *C. albicans* strains (strain 13, 14, and 19) is lower than most of FLC-resistant *C. albicans* strains. Mechanisms of FLC-resistance amongst *C. albicans* isolates are highly variable and often clade specific, the nuances of which are still being elucidated (Perea et al., 2001; Flowers et al., 2015).

To investigate whether the intracellular FLC content can be affected in the presence of other drugs which exhibits synergistic effects with FLC against FLC-resistant *C. albicans* 103 strain, we applied the established approach to detect the intracellular FLC content in the combination of Flos Rosae Chinensis (FRC), BBR, or other herbal extracts derived from the traditional Chinese medicine and FLC. The results showed that the effects of different drugs on intracellular concentration of FLC were diverse. Among these drugs exhibiting synergistic anti-FLC-resistant *C. albicans* activity with FLC, some drugs did increase the intracellular concentration of FLC at the different time points analyzed, some drugs did not affect the intracellular FLC concentration, but some drugs even decreased the intracellular concentration of FLC (data not shown). These data indicated that the mechanisms of these synergistic effects were different from each other. Given the complex and heterogeneity of resistance mechanisms, further investigations are required to explore the exact molecular mechanisms underlying these phenomena. In addition, detection of intracellular FLC content when applying our approach to a combination therapy regimen may be affected by other compounds, such as phosphorus-containing compounds can adsorb onto active sites in the sample flow path, particularly at trace levels, compromising the accuracy of the chromatography. Furthermore, some compounds are more difficult to elute from the column, such as BBR, requiring additional elution time to resolve this. However, combination drugs that affect the stability of FLC have not been encountered.

Taken together, we developed a LC-MS/MS approach, providing a highly accurate, efficient, and reproducible method for quantifying the intracellular concentration of FLC in *C. albicans*. However, the present study has several limitations. First, this LC-MS/MS method was applied to 36 clinical isolated *C. albicans* strains, more clinical isolated *C. albicans* strains would be needed to further investigate this relationship between intracellular drug concentration of FLC-sensitive and FLC-resistant *C. albicans*. Second, the combination therapy regimens can be easily implemented to treat fungal infections (Iyer et al., 2020). Our current easy-to-use detection method may further obtain data on the intracellular drug concentration to explore the underlying mechanism of synergistic antifungal therapy by increasing the intracellular drug content. Third, whether the differences of drug concentration between FLC-sensitive and FLC-resistant *C. albicans* is related to membrane permeability or drug efflux genes needs to be further investigated.

## Data availability statement

The original contributions presented in the study are included in the article/supplementary material, further inquiries can be directed to the corresponding author/s.

## Author contributions

XFW, XJW, YJ, BH, and YC conceptualized the study design. XFW, XJW, TC, and LL conducted experiments. XJW, YQ, and LL wrote the manuscript. YJ, BH, and YC supervised the study and revised the manuscript. All authors contributed to the article and approved the submitted version.

## References

[B1] Alebic-Kolbah T. Modesitt M. S. (2012). Anidulafungin–challenges in development and validation of an LC-MS/MS bioanalytical method validated for regulated clinical studies. *Anal. Bioanal. Chem.* 404 2043–2055. 10.1007/s00216-012-6272-4 22842828

[B2] Arendrup M. C. Patterson T. F. (2017). Multidrug-resistant *Candida*: Epidemiology, molecular mechanisms, and treatment. *J. Infect. Dis.* 216:S445–S451. 10.1093/infdis/jix131 28911043

[B3] Bergeron A. Furtado M. Garofolo F. (2009). Importance of using highly pure internal standards for successful liquid chromatography/tandem mass spectrometric bioanalytical assays. *Rapid Commun. Mass Spectrom.* 23 1287–1297. 10.1002/rcm.4001 19308966

[B4] Berkow E. L. Lockhart S. R. (2017). Fluconazole resistance in *Candida species*: A current perspective. *Infect. Drug Resist.* 10 237–245. 10.2147/idr.S118892 28814889 PMC5546770

[B5] Beste K. Y. Burkhardt O. Kaever V. (2012). Rapid HPLC-MS/MS method for simultaneous quantitation of four routinely administered triazole antifungals in human plasma. *Clin. Chim. Acta* 413 240–245. 10.1016/j.cca.2011.09.042 21996080

[B6] Bongomin F. Gago S. Oladele R. O. Denning D. W. (2017). Global and multi-national prevalence of fungal diseases-estimate precision. *J. Fungi* 3:57. 10.3390/jof3040057 29371573 PMC5753159

[B7] Brown G. D. Denning D. W. Levitz S. M. (2012). Tackling human fungal infections. *Science* 336:647. 10.1126/science.1222236 22582229

[B8] Campitelli M. Zeineddine N. Samaha G. Maslak S. (2017). Combination antifungal therapy: A review of current data. *J. Clin. Med. Res.* 9 451–456. 10.14740/jocmr2992w 28496543 PMC5412516

[B9] de Moraes F. C. Bittencourt S. F. Perissutti E. Frencentese F. Arruda A. M. Chen L. S. (2014). Quantification of dapaconazole in human plasma using high-performance liquid chromatography coupled to tandem mass spectrometry: Application to a phase I study. *J. Chromatogr. B* 958 102–107. 10.1016/j.jchromb.2014.01.053 24705538

[B10] de Oliveira Santos G. C. Vasconcelos C. C. Lopes A. J. O. De Sousa Cartágenes M. D. S. Filho A. Do Nascimento F. R. F. (2018). *Candida* infections and therapeutic strategies: Mechanisms of action for traditional and alternative agents. *Front. Microbiol.* 9:1351. 10.3389/fmicb.2018.01351 30018595 PMC6038711

[B11] Dhasarathan P. Alsalhi M. S. Devanesan S. Subbiah J. Ranjitsingh A. J. A. Binsalah M. (2021). Drug resistance in *Candida albicans* isolates and related changes in the structural domain of Mdr1 protein. *J. Infect. Public Health* 14 1848–1853. 10.1016/j.jiph.2021.11.002 34794907

[B12] Diez L. Martenka E. Dabrowska A. Coulon J. Leroy P. (2005). Assessment of in situ cellular glutathione labeling with naphthalene-2,3-dicarboxaldehyde using high-performance liquid chromatography. *J. Chromatogr. B Anal. Technol. Biomed. Life Sci.* 827 44–50. 10.1016/j.jchromb.2005.02.007 16260378

[B13] Flowers S. A. Colón B. Whaley S. G. Schuler M. A. Rogers P. D. (2015). Contribution of clinically derived mutations in ERG11 to azole resistance in *Candida albicans*. *Antimicrob. Agents Chemother.* 59 450–460. 10.1128/aac.03470-14 25385095 PMC4291385

[B14] Hou J. Deng J. Liu Y. Zhang W. Wu S. Liao Q. (2022). Epidemiology, clinical characteristics, risk factors, and outcomes of candidemia in a large tertiary teaching hospital in Western China: A retrospective 5-year study from 2016 to 2020. *Antibiotics* 11:788. 10.3390/antibiotics11060788 35740194 PMC9220019

[B15] Iyer K. R. Camara K. Daniel-Ivad M. Trilles R. Pimentel-Elardo S. M. Fossen J. L. (2020). An oxindole efflux inhibitor potentiates azoles and impairs virulence in the fungal pathogen *Candida auris*. *Nat. Commun.* 11:6429. 10.1038/s41467-020-20183-3 33353950 PMC7755909

[B16] Johnson M. D. Macdougall C. Ostrosky-Zeichner L. Perfect J. R. Rex J. H. (2004). Combination antifungal therapy. *Antimicrob. Agents Chemother.* 48 693–715. 10.1128/aac.48.3.693-715.2004 14982754 PMC353116

[B17] Kim C. Ryu H. D. Chung E. G. Kim Y. Lee J. K. (2018). A review of analytical procedures for the simultaneous determination of medically important veterinary antibiotics in environmental water: Sample preparation, liquid chromatography, and mass spectrometry. *J. Environ. Manag.* 217 629–645. 10.1016/j.jenvman.2018.04.006 29649735

[B18] Kofla G. Turner V. Schulz B. Storch U. Froelich D. Rognon B. (2011). Doxorubicin induces drug efflux pumps in *Candida albicans*. *Med. Mycol.* 49 132–142. 10.3109/13693786.2010.512022 20818920

[B19] Lu H. Shrivastava M. Whiteway M. Jiang Y. (2021). *Candida albicans* targets that potentially synergize with fluconazole. *Crit. Rev. Microbiol.* 47 323–337. 10.1080/1040841x.2021.1884641 33587857

[B20] Magill S. S. Edwards J. R. Bamberg W. Beldavs Z. G. Dumyati G. Kainer M. A. (2014). Multistate point-prevalence survey of health care-associated infections. *N. Engl. J. Med.* 370 1198–1208. 10.1056/NEJMoa1306801 24670166 PMC4648343

[B21] Pappas P. G. Lionakis M. S. Arendrup M. C. Ostrosky-Zeichner L. Kullberg B. J. (2018). Invasive candidiasis. *Nat. Rev. Dis. Primers* 4:18026. 10.1038/nrdp.2018.26 29749387

[B22] Perea S. López-Ribot J. L. Kirkpatrick W. R. Mcatee R. K. Santillán R. A. Martínez M. (2001). Prevalence of molecular mechanisms of resistance to azole antifungal agents in *Candida albicans* strains displaying high-level fluconazole resistance isolated from human immunodeficiency virus-infected patients. *Antimicrob. Agents Chemother.* 45 2676–2684. 10.1128/aac.45.10.2676-2684.2001 11557454 PMC90716

[B23] Pristov K. E. Ghannoum M. A. (2019). Resistance of *Candida* to azoles and echinocandins worldwide. *Clin. Microbiol. Infect.* 25 792–798. 10.1016/j.cmi.2019.03.028 30965100

[B24] Quan H. Cao Y. Y. Xu Z. Zhao J. X. Gao P. H. Qin X. F. (2006). Potent *in vitro* synergism of fluconazole and berberine chloride against clinical isolates of *Candida albicans* resistant to fluconazole. *Antimicrob. Agents Chemother.* 50 1096–1099. 10.1128/aac.50.3.1096-1099.2006 16495278 PMC1426442

[B25] Quindós G. Marcos-Arias C. San-Millán R. Mateo E. Eraso E. (2018). The continuous changes in the aetiology and epidemiology of invasive candidiasis: From familiar *Candida albicans* to multiresistant *Candida auris*. *Int. Microbiol.* 21 107–119. 10.1007/s10123-018-0014-1 30810955

[B26] Revie N. M. Iyer K. R. Robbins N. Cowen L. E. (2018). Antifungal drug resistance: Evolution, mechanisms and impact. *Curr. Opin. Microbiol.* 45 70–76. 10.1016/j.mib.2018.02.005 29547801 PMC6135714

[B27] Ribeiro de Carvalho R. Chaves Silva N. Cusinato M. Tranches Dias K. S. Dos Santos M. H. Viegas Junior C. (2018). Promising synergistic activity of fluconazole with bioactive guttiferone-a and derivatives against non-albicans *Candida species*. *J. Mycol. Med.* 28 645–650. 10.1016/j.mycmed.2018.07.006 30104135

[B28] Robbins N. Caplan T. Cowen L. E. (2017). Molecular evolution of antifungal drug resistance. *Annu. Rev. Microbiol.* 71 753–775. 10.1146/annurev-micro-030117-020345 28886681

[B29] Rocha M. F. G. Bandeira S. P. De Alencar L. P. Melo L. M. Sales J. A. Paiva M. A. N. (2017). Azole resistance in *Candida albicans* from animals: Highlights on efflux pump activity and gene overexpression. *Mycoses* 60 462–468. 10.1111/myc.12611 28295690

[B30] Różalska B. Sadowska B. Budzyńska A. Bernat P. Różalska S. (2018). Biogenic nanosilver synthesized in metarhizium robertsii waste mycelium extract - as a modulator of *Candida albicans* morphogenesis, membrane lipidome and biofilm. *PLoS One* 13:e0194254. 10.1371/journal.pone.0194254 29554119 PMC5858827

[B31] Tang J. Wei H. Liu H. Ji H. Dong D. Zhu D. (2010). Pharmacokinetics and biodistribution of itraconazole in rats and mice following intravenous administration in a novel liposome formulation. *Drug Deliv.* 17 223–230. 10.3109/10717541003667822 20210560

[B32] Tome M. Zupan J. Tomičić Z. Matos T. Raspor P. (2018). Synergistic and antagonistic effects of immunomodulatory drugs on the action of antifungals against *Candida glabrata* and Saccharomyces cerevisiae. *PeerJ* 6:e4999. 10.7717/peerj.4999 29915703 PMC6004109

[B33] Van De Steene J. C. Lambert W. E. (2008). Comparison of matrix effects in HPLC-MS/MS and UPLC-MS/MS analysis of nine basic pharmaceuticals in surface waters. *J. Am. Soc. Mass Spectrom.* 19 713–718. 10.1016/j.jasms.2008.01.013 18343682

[B34] Wadsworth J. M. Milan A. M. Anson J. Davison A. S. (2017). Development of a liquid chromatography tandem mass spectrometry method for the simultaneous measurement of voriconazole, posaconazole and itraconazole. *Ann. Clin. Biochem.* 54 686–695. 10.1177/0004563216686378 27941128

[B35] Wiederhold N. P. (2017). Antifungal resistance: Current trends and future strategies to combat. *Infect. Drug Resist.* 10 249–259. 10.2147/idr.S124918 28919789 PMC5587015

[B36] Xiang J. Yu Q. Liang M. Z. Qin Y. P. Nan F. (2018). [Determination of voriconazole in human plasma and its bioequivalence by HPLC-MS/MS]. *Sichuan Da Xue Xue Bao Yi Xue Ban* 49 102–106. 29737099

[B37] Xu Y. Lu H. Zhu S. Li W. Q. Jiang Y. Y. Berman J. (2021). Multifactorial mechanisms of tolerance to ketoconazole in *Candida albicans*. *Microbiol. Spectr.* 9:e0032121. 10.1128/Spectrum.00321-21 34160280 PMC8552639

[B38] Zacchino S. A. Butassi E. Cordisco E. Svetaz L. A. (2017). Hybrid combinations containing natural products and antimicrobial drugs that interfere with bacterial and fungal biofilms. *Phytomedicine* 37 14–26. 10.1016/j.phymed.2017.10.021 29174600

[B39] Zgoła-Grześkowiak A. Grześkowiak T. (2013). Application of dispersive liquid-liquid microextraction followed by HPLC-MS/MS for the trace determination of clotrimazole in environmental water samples. *J. Sep. Sci.* 36 2514–2521. 10.1002/jssc.201300271 23720393

[B40] Zhang H. Wang X. Qian M. Wang X. Xu H. Xu M. (2011). Residue analysis and degradation studies of fenbuconazole and myclobutanil in strawberry by chiral high-performance liquid chromatography-tandem mass spectrometry. *J. Agric. Food Chem.* 59 12012–12017. 10.1021/jf202975x 21967215

[B41] Zimmer D. (2014). New US FDA draft guidance on bioanalytical method validation versus current FDA and EMA guidelines: Chromatographic methods and ISR. *Bioanalysis* 6 13–19. 10.4155/bio.13.298 24256335

